# A multi-species repository of social networks

**DOI:** 10.1038/s41597-019-0056-z

**Published:** 2019-04-29

**Authors:** Pratha Sah, José David Méndez, Shweta Bansal

**Affiliations:** Department of Biology, Georgetown University, Washington, DC USA

**Keywords:** Ecological epidemiology, Network topology, Data publication and archiving, Behavioural ecology

## Abstract

Social network analysis is an invaluable tool to understand the patterns, evolution, and consequences of sociality. Comparative studies over a range of social systems across multiple taxonomic groups are particularly valuable. Such studies however require quantitative social association or interaction data across multiple species which is not easily available. We introduce the Animal Social Network Repository (ASNR) as the first multi-taxonomic repository that collates 790 social networks from more than 45 species, including those of mammals, reptiles, fish, birds, and insects. The repository was created by consolidating social network datasets from the literature on wild and captive animals into a consistent and easy-to-use network data format. The repository is archived at https://bansallab.github.io/asnr/. ASNR has tremendous research potential, including testing hypotheses in the fields of animal ecology, social behavior, epidemiology and evolutionary biology.

## Abstract (summary)

**Table:** 

Design Type(s)	species comparison design • network analysis objective • data integration objective
Measurement Type(s)	social interaction measurement
Technology Type(s)	digital curation
Factor Type(s)	Taxon • experimental condition • geographic location
Sample Characteristic(s)	Gasterosteus aculeatus • Poecilia reticulata • Hirundo rustica • Branta leucopsis • Gallus gallus • Haemorhous mexicanus • Zonotrichia atricapilla • Acanthiza • Philetairus socius • Aves • Camponotus fellah • Camponotus pennsylvanicus • Bolitotherus cornutus • Elephas maximus • Papio cynocephalus • Desmodus rotundus • Myotis sodalis • Bison bison • Bos taurus • Tursiops truncatus • Mirounga angustirostris • Crocuta crocuta • Macropus giganteus • Trichosurus cunninghami • Ateles geoffroyi • Macaca fuscata • Macaca mulatta • Macaca radiata • Macaca tonkeana • Pan paniscus • Pan troglodytes • Papio papio • Saguinus fuscicollis • Saguinus mystax • Trachypithecus johnii • Alouatta guariba • Brachyteles arachnoides • Sapajus apella • Cercopithecus campbelli • Colobus guereza • Erythrocebus patas • Macaca arctoides • Macaca assamensis • Procyon lotor • Ovis canadensis • Ateles hybridus • Microtus agrestis • Equus • Gopherus agassizii • Tiliqua rugosa

Machine-accessible metadata file describing the reported data (ISA-Tab format)

## Background & Summary

Network analysis is a central approach in several basic and applied research areas of ecology and evolutionary biology, including behavioral ecology, epidemiology, spatial ecology, and social evolution^1^. Recently, researchers have demonstrated the utility of network analysis in explaining the transmission of social information in animal groups^2,3^, evolution of cultural behavior^4^, epidemiological consequences of group substructure^5^, mechanisms of infectious disease transmission in animal groups^5–7^, and emergence of collective behavior^8^. Network analysis leverages detailed social association, interaction and movement data and allows for the incorporation of heterogeneity at the individual scale to explain population level processes, as well as the ability to objectively quantify the organization of social interactions and dynamics of group behavior.

Recent advances in computational power and technological tools, such as proximity loggers and radio-frequency identification, have facilitated the collection of association and interaction data^9^. And with the open science movement gaining steady momentum^10^, a culture of making research data and experimental methods publicly available and transparent has unleashed valuable social network data for use by all researchers. For the first time, there is thus an opportunity to carry out comparative network studies across multiple species to identify general patterns and generate broad principles^5^. However, such studies are currently challenging due to differences in data collection methods, a lack of standardized formats for published data, and the absence of a centralized data repository^11^. While such repositories exist for human interaction data^12–15^, there is a gap for social network datasets across multiple taxonomic groups.

Here, we introduce the Animal Social Network Repository (ASNR), which fills this gap by providing access to networks of social associations or interactions across multiple taxonomic groups organized in a consistent network file format. The repository provides opportunities for: (a) field biologists to generate preliminary hypotheses and plan for data collection (including the resolution, duration and quality) required to test their hypotheses; (b) empiricists to evaluate the effects of data collection methods on observed network properties and characteristics; (c) behavioral ecologists to compare social structures within and across broad taxonomic groups; (d) network scientists to analyze the patterns and function of dynamic networks; (e) evolutionary biologists to understand the drivers for the emergence of disparate network structure across different species; and (f) and disease ecologists to understand the eco-epidemiological implications of the evolved network structure. The repository thus has tremendous research potential in the fields of ecology, epidemiology, evolution, behavior, and beyond.

## Methods

### Selection methodology

The data repository was collated by reviewing published literature and popular data repositories, including the Dryad Digital Repository, Harvard Dataverse and figshare, for social network datasets associated with peer-reviewed publications. We used terms such as “social network”, “social structure”, “interaction network”, “animal networks”, “network behavior”, among others, to perform our electronic search. Additional network datasets were acquired through convenience sampling by directly contacting the authors of published studies without open data. Only studies on non-human species were included, and studies reporting non-interaction or non-association networks such as biological networks or food-web networks were excluded. Studies that did not include enough information for networks to be re-created were also excluded. By reviewing the quality of the remaining published datasets (see Technical Validation), a total of 790 social networks from more than 45 animal species and 18 taxonomic orders were selected for the data repository.

## Data Records

The social network files from this study are available through the ASNR website (https://bansallab.github.io/asnr) and Harvard Dataverse^16^. The Harvard Dataverse version is a snapshot of the dataset to match the information given in this Data Descriptor. The file ‘Network_summary_master_file.csv’ at Harvard Dataverse summarizes the dataset. The ASNR website serves as a dynamic platform, where new network datasets can be curated and added. For easy access to the datasets, the repository is organized into sections each representing a unique taxonomic group. Each section further consists of a set of social networks which were collected together with the same sampling method. The datasets are uniquely identified with the animal species first, followed by the association or interaction type and ending with the edge weight criteria (weighted or unweighted). In cases where multiple networks are available within each dataset, each social network is assigned a unique identifier. A readme file is also included with each dataset that summarizes structural features of the networks and provides information on the original source.

Each network dataset is provided in the GraphML format^17^. GraphML is a flexible and convenient XML format for storing network information. It supports unweighted, weighted, undirected and directed networks and allows for the definition of node and edge attributes.

## Technical Validation

Our validation process consisted of data-type and constraint validation, structural validation, and cross-reference and ecological validation. All data collection and validation steps were carried out by two co-authors (PS and JM).

### Data-type and constraint validation

The first step involved quality checks to ensure that the original data contained enough information to enable reconstruction of social network(s). All datasets were acquired in electronic format in one of the following four network data structures: edgelists, adjacency matrices, adjacency lists or group membership dataframes. All data was classified into nodes, edges or attribute data. All node ids were verified to be of the same type (e.g. integer or string). All edges were verified to be between nodes in the node list, or were added as nodes to the node list. All attribute data was verified to correspond to an existing node or edge.

### Structural validation

We next validated the structural integrity of the network described in the original data-source by removing all edges that connected any node to itself (i.e. self loops). Any duplicate edges were also removed. Individuals with no edges (i.e. isolated nodes) were not removed from the network.

The ASNR currently only contains static networks. Thus, multiple associations or interactions reported between the same node pair at different time-points were replaced with weighted edges, with weights representing the association/interaction frequency.

### Cross-reference and ecological validation

For detecting errors in the data mining and GraphML conversion process, we calculated network summary statistics (e.g. number of nodes, number of edges, clustering coefficient) for each network and cross-checked them against the network description in the original publication. The structures of each converted network file were also cross-checked to ensure consistency within the ecological context of data collection. For example, networks of the same group of individuals of a species that were collected over mating vs. non-mating season are expected to differ in terms of their network densities.

### Data characterization

In the sections below, we characterize the phylogenetic and geographical distribution, data collection methodology, and structural similarity of the networks included in the repository.

### Phylogenetic and geographic distribution

**Fig. 1:**
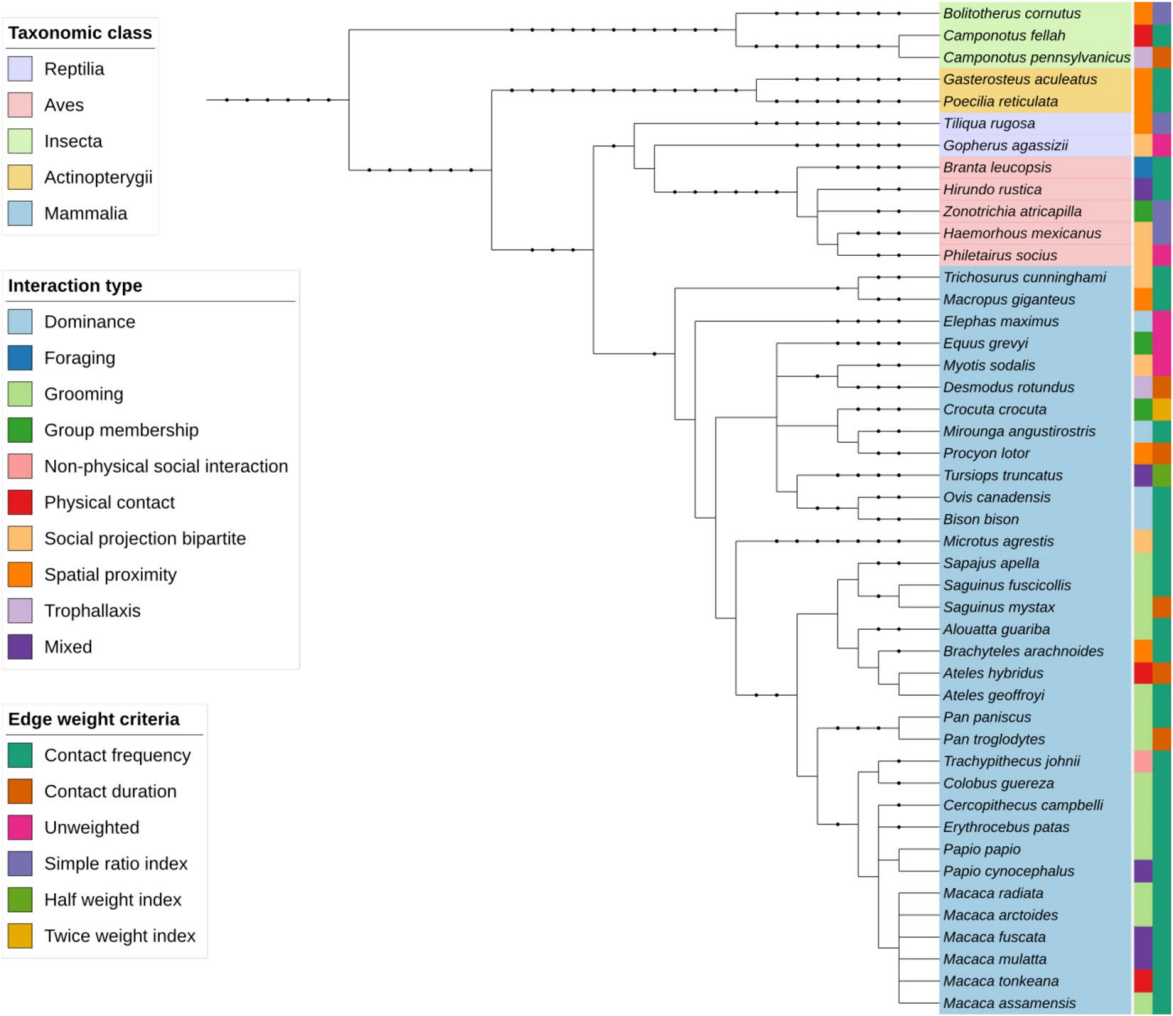
Phylogenetic distribution of non-human species included in the Animal Social Network Repository (ASNR). The first color strip includes the species’ scientific name, and is color coded according to the taxonomic class. The second color strip is coded according to the social interaction quantified in the network, and the third color strip is coded according to the weighting criteria of the network edges. Datasets that had multiple species or with unspecified species name were not included in the figure.

The phylogenetic distribution of the taxonomic groups currently included in the repository is shown in Fig. 1. While mammals are the most studied taxa, social networks from other taxa including reptiles, birds, insects, and fish also exist.

**Fig. 2:**
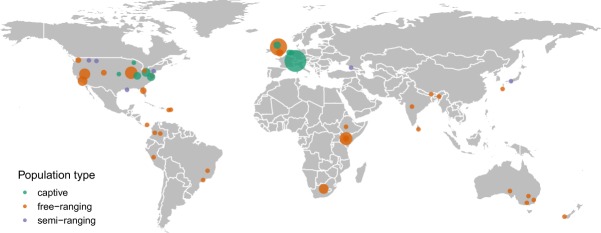
Geographical distribution of the social networks included in ASNR. The points indicate the geographical location where data for each social network was collected. The point size is proportional to the number of social networks collected at each location. Point color denotes whether the monitored animal populations were captive, semi-ranging or free-ranging.

The geographical locations where data for each social network were collected is shown in Fig. 2. The United States contributes the largest number of studies and the repository contains data from Central and South America, Europe, Africa, Asia and Australia. Additionally, most studies are in free-ranging populations.

### Behavioral types

The behavioral data span a range of social associations from direct physical contacts such as grooming and trophallaxis to indirect interactions such as spatial proximity and association (Fig. 1).

Additionally, contact intensity were distributed across six categories–unweighted (i.e., all edges have weight equal to one), contact frequency, contact duration, simple ratio index^18^, twice weight index^19^, and half weight index^18^ (Fig. 1).

### Data collection methodology

**Fig. 3:**
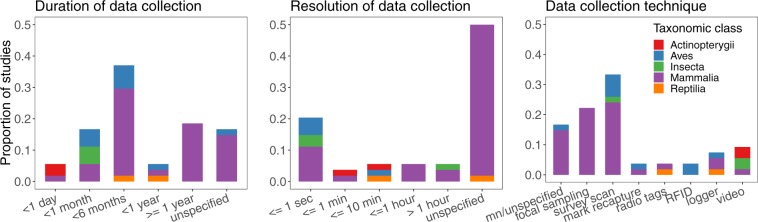
Duration, time resolution and technique of data collection of social networks included in the repository. mn = manual, RFID = radio-frequency identification.

Figure 3 summarizes the methodology and data collection techniques described in original data sources that were used to collect the networks. We highlight that studies rely on a variety of data collection methodologies and timescales, reflecting empirical constraints and the disparate scientific purposes of each study. It is important that future comparative studies take these differences into account^11^.

### Assessing network structure

We used the Python NetworkX package^20^ to examine the structural properties of the social networks associated with each species. We calculated the following structural properties for each social network in the repository: total nodes, total edges, network density, network average degree, degree heterogeneity, degree assortativity, average clustering coefficient (unweighted and weighted), transitivity, average betweenness centrality (unweighted and weighted), average clustering coefficient (weighted and unweighted), Newman modularity, maximum modularity, relative modularity, group cohesion, and network diameter. These network metrics are defined in Table 1.

**Table 1:** Structural properties of the networks described in ASNR.

Network measure	Description
Total nodes	Total number of individuals present in the social network.
Total edges	Total pairwise social associations/interactions recorded in the network.
Network density	Sum of the edges divided by the total number of possible edges in the social network.
Network average degree	The average number of edges connecting each node.
Degree heterogeneity	Coefficient of variation (CV) in the degree distribution, measured as the standard deviation in degree divided by the mean degree.
Degree assortativity	The tendency of social partners to have a similar degree. Measured as the correlation coefficient between the degrees of neighboring nodes.
Average clustering coefficient (unweighted)	The average of clustering coefficient of nodes in the network. Clustering coefficient of a node is measured as the fraction of all possible triangles through the node that exist in the network, and indicates the propensity of its neighboring nodes to interact with each other.
Average clustering coefficient (weighted)	Similar metric as the average clustering coefficient, but taking edge weights into account as described in^21^.
Transitivity	Fraction of all possible triangles present in the social network. This metric provides a network-level measure of the presence of cliques (triangles) as opposed to average clustering coefficient that summarizes clustering at node-level.
Average betweenness centrality (unweighted)	Average betweenness centrality of all nodes present in the network. Betweenness centrality is a measure of how central a node is in the network, and is defined as the number of shortest path that go through the focal node in the network. Nodes in a social network with high average betweenness centrality have a greater tendency to occupy socially central positions.
Average betweenness centrality (weighted)	Average betweenness centrality of the network taking edge weights into account.
Newman modularity	A common measure to estimate the strength of subdivision in networks^22^. Higher values of Newman modularity indicate stronger subdivisions of social networks. Newman modularity was estimated using the Louvain algorithm^23^
Maximum modularity	The highest possible modularity achieved when all individuals in a group only interact with each other and no edges are present between different groups^5^.
Relative modularity	Normalized Newman modularity as described in^5^.
Group cohesion	Proportion of the total associations or interactions that occur within the groups (modules) identified using the Lovain method^23^. Groups are defined as a subset of individuals that preferentially associate/interact with each other than the rest of the individuals present in the network. High group cohesion indicates higher individual preferences to interact with members of own module.
Network diameter	The maximum shortest distance (in terms of the number of hops) between any pair of nodes in the largest connected component of a network. Information typically spreads faster in networks with a smaller diameter.

**Fig. 4:**
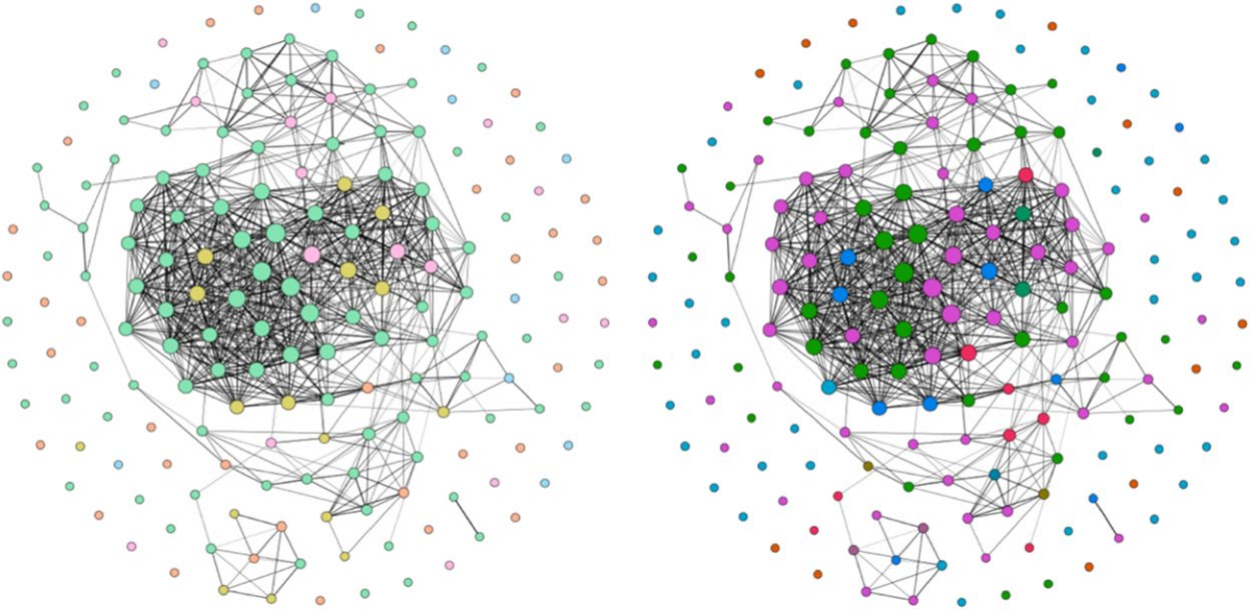
Graphical representation of similarity of networks based on six network metrics–degree heterogeneity, network density, average clustering coefficient, degree assortativity, betweenness centrality and relative modularity. Each node in the network represents a unique social group of an animal species, and an edge between two nodes demonstrates the similarity of their network structure. If a social group contained more than one network (for example, snapshots of a temporal network), an average value was calculated for each network metric. A z-score of each network metric was calculated. Two social groups were considered to be structurally similar (and connected by edges) if they were within one standard deviation of each other in the z-score distribution of all six network metrics. The figures on the left and right are identical except for node colors: (left) node colors indicate taxonomic classes. Green - Mammalia, orange - Aves, pink - Actinopterygii, yellow - Insecta and blue - Reptilia. (right) Node colors indicate type of interaction represented as edges. Pink - spatial proximity, green - grooming, light blue - social projection bipartite, orange - group membership, dark blue - physical contact, red - dominance interaction, dark green - trophallaxis, brown - foraging, purple - non physical social interaction, teal - overall mix.

In Fig. 4 we capture the structural similarity between the social networks included in the repository. Social networks of mammals tend to cluster together, although some structural overlap also exists with the social networks of insects and fish. Social networks that describe spatial proximity, physical contact or grooming interactions between individuals tend to be structurally similar.

## Usage Notes

There are several open-source network libraries that can be used to analyze and visualize the networks provided in GraphML format at ASNR. Examples of network analysis and visualization softwares include NetworkX in Python, igraph in R, Cytoscape, yEd and Gephi.

## ISA-Tab metadata file

Download metadata file

## Data Availability

All code for data characterization has been written in *Python* using the Networkx package. The code is open source at https://github.com/bansallab/asnr/.
